# Optical bleaching front in bedrock revealed by spatially-resolved infrared photoluminescence

**DOI:** 10.1038/s41598-019-38815-0

**Published:** 2019-02-22

**Authors:** E. L. Sellwood, B. Guralnik, M. Kook, A. K. Prasad, R. Sohbati, K. Hippe, J. Wallinga, M. Jain

**Affiliations:** 1 Soil Geography and Landscape group & Netherlands Centre for Luminescence dating, Wageningen University, 6708PB Wageningen, The Netherlands; 2 Centre for Nuclear Technologies, Technical University of Denmark, DK, 4000 Roskilde, Denmark; 3 The Nordic Laboratory for Luminescence Dating, Department of Geoscience, Aarhus University, DK, 4000 Roskilde, Denmark; 4 CAPRES A/S & DTU Nanotech, Diplomvej 373, 2800 Kgs Lyngby, Denmark; 5 Laboratory of Ion Beam Physics, 8093 Zürich, Switzerland; 6 Institute of Geological Sciences, Freie Universität Berlin, 12249 Berlin, Germany

## Abstract

Optically stimulated luminescence (OSL) dating of sediment, based on the accumulation of trapped charge in natural crystals since their last exposure to daylight, has revolutionised our understanding of the late Quaternary period. Recently, a complementary technique called luminescence rock surface dating (RSD), which uses differential spatial eviction of trapped charges in rocks exposed to daylight, has been developed to derive exposure and burial ages, and hard-rock erosion rates. In its current form, the RSD technique suffers from labour intensive sample preparation, uncertainties in the depth and dose rate estimates, and poor resolution of the luminescence-depth profile. Here, we develop a novel, 2D luminescence imaging technique for RSD of large rock slabs (3 × 5 cm) to overcome these challenges. We utilize the recently discovered infrared photoluminescence (IRPL) signal for direct, non-destructive imaging of the luminescence-depth profile in a sub-aerially exposed granitic rock, with an unprecedented spatial resolution of ~140 µm. We further establish a correlation between luminescence and geochemistry using micro X-ray fluorescence (µXRF) spectroscopy. Our study promises a substantial advancement in luminescence imaging and paves the path towards novel applications using 2D dating, micro-dosimetry in mixed composition samples, and portable instrumentation for *in-situ* luminescence measurements.

## Introduction

The surface of Earth evolves dynamically in response to changes in climate, sea level, tectonics and land use. Studying landscapes is important both for understanding the forcing and feedback mechanisms in different components of the Earth system, and for developing strategies for future sustainable land use. Measurements of the rates of processes that induce changes in the landscape (e.g. erosion, uplift, exhumation, accumulation, etc.)^1–4^, are critical for obtaining such an understanding. Despite rapid developments in quantitative geomorphology over the past decades^5^, determining time-averaged process rates over timescales of hundreds to tens of thousands of years, and on sub-centimetre spatial scales is particularly challenging because of a lack of appropriate methods. Recently, optically stimulated luminescence (OSL)^6^ has been adapted for low temperature thermochronometry^7,8^ and rock surface dating (RSD)^9^, to provide access to process information on such time scales in a wide range of environmental settings. In contrast to cosmogenic nuclides (CN) dating, the luminescence RSD technique evaluates changes over much finer spatial (10^−4^ to 10^−2^ m)^10,11^ and temporal scales (10° to 10^5^ years) scales^9,11^, offering the potential to investigate the effects of local-scale topography, microclimate, and lithology on landscape evolution. Inheritance effects which can be especially problematic in CN dating of young samples^12^, are usually identifiable in OSL RSD from the evaluation of the signal-depth profile; it enables the identification and, under certain circumstances, dating of multiple burial and exposure events recorded in the shape of the profile^13–15^. OSL RSD has been used in a wide range of challenging applications such as dating of rock art^16,17^ and archaeological artefacts^9,13–15,18^, bedrock and boulder surfaces in periglacial^4^ and glacio-fluvial environments^19^, and estimation of rock erosion rates^1^ and transport durations^20^. Similarly, the method holds promise for dating mass-wasting events^17^ and determining fault slip rates and soil turnover rates. OSL RSD has thus the potential to date events in rock histories, thus, filling the gap in techniques between the traditional CN methods on one hand and the modern tracer methods on the other^2,21^.

OSL RSD dating is based on the principle that attenuation of daylight flux through an exposed rock results in an S-shaped luminescence-depth profile^22,23^; this is due to differential eviction of trapped charges by sunlight, with depth^11^. The bleaching front refers to the depth at which luminescence reaches about 50% of the saturation level, and is a function of exposure duration (i.e. age), photo-ionisation cross-section of the luminescent minerals, spectrum and flux of the incident daylight, dose rate, and the rock opacity^10,11,22,24^. The exposure age can then be determined by fitting an age model^1,9^ where the key unknown parameter (dretrapping rate) is quantified by empirical calibration. The bleaching front paces logarithmically with time and reaches a steady state in about 0.1 Ma^11^; beyond which only minimum exposure ages can be inferred^11^. The bleaching front is critical for estimating the reliability of RSD of pebbles and cobbles in challenging deposits such as glacial tills and moraines, floods, archaeological artefacts, etc., where optical resetting prior to deposition is uncertain. Here the bleaching front can be used to (a) confirm whether luminescence clock was fully reset at or close to the surface^17^, and (b) possibly determine pre-burial exposure duration giving additional process information^9,17^.

**Figure 1:**
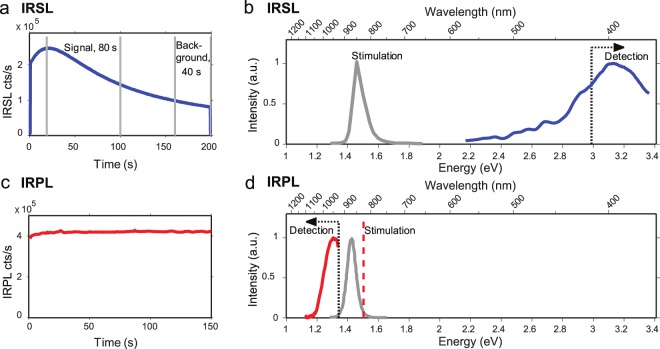
(**a**) Example of a pIR-IRSL_225_ decay curve suffering from thermal lag, observed seen as initial rise in the signal, due to a delay in the rock slice reaching equilibrium with the heater (measurement) temperature. (**b**) IRSL stimulation and emission spectra taken from Prasad *et al*.^38^. The 350–415 nm filter cut-off used in IRSL data collection is shown as the dotted line. The LED stimulation spectrum is shown as the red curve (**c**) Example of the time dependence of IRPL signal measured from our sample. The signal shows steady state after a slight initial rise due to laser stabilisation. Signal integration limit can be chosen as desired to achieve high signal-to-noise ratio. d) IRPL measurement configuration. The grey and the red curves show the IRPL excitation and emission spectra, taken from Prasad *et al*.^38^, respectively. An 830 nm laser (dashed line), cleaned up using an 850 nm short-pass filter, was used for excitation. IRPL was measured using a 950 Δ50 nm band pass filter in front of the EM-CCD. The laser is blue shifted with respect to the peak of the excitation spectrum; this was deliberately chosen to reduce the breakthrough in the detection and thus enhance the signal-to-noise ratio.
(iv) Uncertainties due to mineralogical heterogeneity: local variations in the light attenuation due to opaque minerals^25^ and dose rates can lead to scatter in luminescence-depth profiles. A spatial correlation of luminescence intensity and mineralogy along the depth dimension is necessary to understand and tackle such scatter.
(v) Inefficient and expensive: Often many rocks have unsuitable luminescence characteristics (opaque and/or insensitive minerals) or have not had any exposure to light (e.g. in case of buried cobbles in floods or moraines). As a consequence, large amounts of samples are often collected, with only a small number of cores providing usable luminescence-depth profile.

Although, highly promising, the OSL RSD sample preparations and measurements are highly cumbersome and inefficient (only few rocks may give a usable profile). Small cores (~1 cm in diameter, 3–4 cm in depth) are drilled perpendicular to the rock surface of interest, and further sliced into a sequence of thin (~1 mm) discs^10^ to measure the luminescence and construct a luminescence-depth profile. Outlined below are the main concerns with the current OSL RSD technique:

(i) Data resolution: a typical OSL-depth profile consists of ~20 data points (slices). The data resolution is limited by material loss between slices due to the thickness of the cutting blade (typically 0.3 mm); this affects the precision of the fitted model parameters. Furthermore, the effective light attenuation coefficient (µ, see section 6)^10,11,13,25^ is constrained by only three to four data points in the vicinity of the bleaching front, resulting in large uncertainties in the exposure age estimate.
(ii) Luminescence models: mathematical models used in RSD are based on trapping and detrapping of electrons, while the OSL (and TL) signal used in RSD involves both trapped electrons and trapped holes. In the case of feldspar, which are widely used in OSL RSD, the transport and electron-hole recombination process can by highly complex^26–28^. Therefore, it is desirable to have experimental methods which directly measure the trapped electron population for compliance between the data and the mathematical models^10,13,29^.
(iii) Uncertainties due to coring and slicing: There is frequent mechanical breakage of both the cores and the individual slices during drilling and slicing. About 10–15% of the mass is ground away by the diamond blade. Due to vibrations and precession/flexing of the blade and/or hardness variations within the core, the slices may be uneven, have varying thicknesses, and cut non-perpendicular to the core axis. These factors lead to uncertainties in depth estimates, and random inter-slice scatter in OSL intensity, e.g. due to varying thermal lag^30,31^ (Fig. 1a) and backscatter^32^ during beta irradiation.

One solution to these challenges is high-resolution spatially-resolved measurement of OSL (HR-OSL)^33–35^ to capture the entire luminescence-depth profile in a single image, avoiding the need for coring and slicing. Although 2D mapping of trapped charge is common in medical and industrial dosimetry (e.g. ESR^36^, OSL and magnetic resonance imaging^37^), it is not routinely practiced in luminescence dating since natural minerals like quartz or feldspars have orders of magnitude lower OSL sensitivity (luminescence intensity per unit mass per unit dose, kg^−1^Gy^−1^) compared to the artificial dosimeters. The challenge becomes even more severe for mapping large areas (i.e. cm scale OSL-depth profile) relevant to RSD.

In this study, we develop a novel solution for imaging bleaching fronts in rocks using high-resolution (HR) mapping of trapped electrons by infrared photoluminescence (IRPL)^38^. We demonstrate our new spatially resolved HR-IRPL method on a known-age glacially polished rock from Switzerland^39^. The entire luminescence depth profile is captured in a single image of a large (3 × 5 cm) granite rock section cut perpendicular to the natural light-exposed surface (see section 6 for details). The IRPL-depth profile is then compared to conventional profiles obtained by coring/slicing and measurements using post IR-IRSL at 225 °C (pIR-IRSL_225_)^37,38^. We further demonstrate the possibility of quantitative elemental mapping of the main granitic constituents via micro X-ray fluorescence (µXRF), correlating it with IRPL intensity and report on the bleaching characteristics of the HR-IRPL signal for rock surface dating. Both the spatial sample scale and the data resolution in this study are unprecedented, and mark a paradigm shift in imaging applications using natural dosimeters.

## Non-destructive optical imaging of trapped charge

The measurement of spatially-resolved (SR) luminescence in rocks was first introduced about two decades ago^40,41^. Over the years photographic film^42,43^, photon counters^44^, and charge-coupled device (CCD) and EM-CCD (electron multiplying CCD) cameras have been used to map OSL and thermoluminescence (TL) signals^43,45,46^. In particular, the advent of single photon counting detectors such as EM-CCDs has enabled an improvement in the resolution of SR-OSL and SR-TL images down to 50 µm/pixel^47^ raising possibilities for 2D dosimetry^34,45,48^ by combining luminescence maps with geochemical data obtained from scanning electron microscopes (SEM) or from X-ray florescence (XRF). However, there are two technical challenges in applying OSL and TL imaging to RSD. Firstly, the imaging area should be comparable to the depth over which the bleaching profile develops. The OSL-depth profiles are typically 3–5 cm in length, and thus an adequately large imaging area should be >3 × 3 cm in size. At such spatial scales, it is challenging to achieve uniform high power illumination required for OSL (e.g., ~200 mW cm^−2^ at 880 nm) or uniform heating for TL, preheating and elevated temperature OSL. Secondly, the increase in imaging size is at the cost of spatial resolution because of the reduction in the light collection efficiency. Even if one were to design a laser scanning system to achieve high power illumination and high sensitivity, the primary restriction (in addition to cross-talk) on spatial resolution comes from the sample emission itself. The OSL/TL emission is based on a fundamentally destructive readout mechanism, i.e. typically one trapped electron gives rise to one luminescence photon by recombination with a trapped hole. In practice, there is less than one emitted photon per detrapped electron because of the presence of non-radiative recombination pathways in OSL. This limitation on signal emission restricts high resolution OSL and TL mapping in natural materials.

Recently, Prasad *et al*.^38^ reported a method for non-destructive, repeatable readout of dosimetric information in feldspar using infra-red photoluminescence (IRPL). These authors used 885 nm (or 842 nm) laser for resonant excitation of trapped electrons leading to a Stokes shifted luminescence emission at 955 nm (1.3 eV) (Fig. 1d) within the principle trap^49,50^ in feldspar. Unlike the OSL or TL processes, IRPL does not involve electron-hole recombination; the signal arises from repeated transitions of the trapped electrons between the defects’ excited and ground states^38^. Thus, while a single trapped electron can at the most produce one photon in OSL/ TL, it can produce millions of photons in IRPL, thereby increasing luminescence emission by many orders of magnitudes. As shown by Prasad *et al*.,^38^ at cryogenic measurement temperatures IRPL is entirely non-destructive. At room temperature a fraction of trapped electrons (with nearby holes) undergo recombination to produce IRSL (Fig. 1a), seen as an initial decrease in the IRPL intensity followed by a steady state IRPL. The electrons that eventually recombine to produce IRSL, emit a large number of IRPL photons through excitation-relaxation prior to recombination^26,27^, in addition to those that are stable and give steady-state IRPL. Since IRPL measurements can be carried out at very low illumination power due to high sensitivity of the signal^51^ even at room temperature the IRPL signal appears to be steady-state (i.e. non decaying, Fig. 1c) at the time scale involved in a typical measurement. The steady state behaviour of IRPL implies that the signal can be integrated over long durations to achieve the counting statistics (i.e. increase the signal-to-noise ratio) necessary for high resolution imaging and precise dating applications (Fig. 1c). IRPL is more suitable than OSL for the mathematical models of RSD since it directly measures the trapped electrons described in the models. In the following sections we explore the suitability of IRPL for direct mapping of the optical bleaching front.

## Results

### High resolution imaging using infra-red photoluminescence

**Figure 2:**
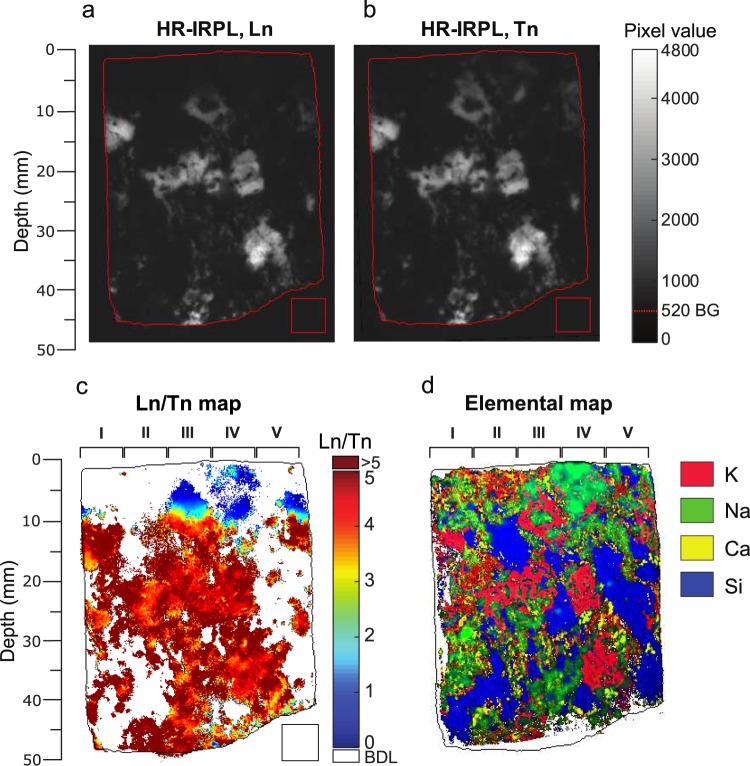
(**a**) HR-IRPL image of natural (L_n_) daylight bleached signal with the intensity scale as shown in the legend. The darker region in the top ~9 mm of the slab suggests daylight bleaching of the IRPL. (**b**) HR-IRPL image after slab was irradiated with 2.5 kGy (T_n_). The top 9 mm of the slab now shows increased IRPL emission compared to the L_n_. The slab edge is marked in red in both (**a**,**b**). The red square area marked below the slab was used to evaluate the background arising mainly from the scattered light; the highest pixel value of 520, used for background subtraction in further image analysis, is indicated as the dashed red line on the greyscale bar. Note that (**a**,**b**) are shown without background subtraction. (**c**) L_n_/T_n_ map, with the greatest intensity changes shown by the blue area in the upper region of the slab. The BDL (below detection level) in the colour scale bar refers to pixel values ≤ 0 in the background subtracted L_n_ and T_n_ images. Five subdivisions of the rock slab are labelled (I–V), used for creating the five depth profiles. (**d**) µXRF map showing K, Na, Ca and Si distributions in the same rock slab used for the HR-IRPL measurements.

The HR-IRPL measured from the rock slice cut from the granite sample (L_n_) was obtained using 830 nm (1.49 eV) laser excitation, after a pre-heat at ~250 °C for 5 minutes (Fig. 2a). The regenerated HR-IRPL (T_n_) after delivering a gamma dose of 2.5 kGy and an identical preheat to the same slice is shown in Fig. 2b. Unlike OSL, the IRPL signal is not reset during measurement. Therefore, a large regeneration dose was chosen to achieve full occupancy of the principle trap, thus, enabling appropriate normalisation of L_n_ signal for all depths. A lower regeneration dose would have led to distortion of IRPL-depth profile since trapping efficiency for the regeneration dose will be different at different depths, a function of the pre-existing occupancy produced during daylight bleaching.

The natural (L_n_) and the regenerated (T_n_) HR-IRPL images appear similar except in the top ~9 mm where L_n_ intensity systematically increases with depth of (Fig. 2a). In contrast to L_n_, the top 9 mm shows a brighter IRPL in the Tn image (Fig. 2b) suggesting that the traps in this region were likely emptied during the daylight exposure of the rock. We also mapped the IRSL signal from the same rock after a second 2.5 kGy gamma dose to compare the signal sensitivity and image resolution with the IRPL signal (these data are shown in Appendix A.1). As expected, the SR-IRSL images had a significantly lower signal intensity, low signal-to-noise ratio and blurry mineral boundaries in comparison to the HR-IRSL images. Hence SR-IRSL was not considered further in this study.

The ratio of the natural over the regenerated HR-IRPL images (L_n_ /T_n_) is shown in Fig. 2c. As expected, L_n_/T_n_ increases systematically from ~0 in the daylight bleached upper part of the slab (blue regions), towards saturated values of ~5 and above (red regions) in the middle and bottom areas of the slab. About 39% of the pixels within the slab had IRPL signals below the detection limit (BDL). These non IRPL emitting regions are attributed to the presence of dark or non-luminescent minerals at the rock slabs surface; such regions are unlikely to be improved by longer integration time since signal intensity is similar to the background level. A small proportion of pixels (~2%) had rather high L_n_/T_n_ ratios (up to 5.6 × 10^4^). Visual inspection confirmed that such values only occur at sharp intensity gradients along mineral boundaries throughout the slab. These values arise either from slight misalignment of pixels across the two images thus inaccurately superimposing non-luminescent and luminescent regions, or potentially from high concentrations of trapping sites which can occur at mineral boundaries^52^. An arbitrary threshold was set at L_n_/T_n_ = 5 to reject any pixels with higher values.

Elemental maps of the previously measured slab were obtained using µXRF, with a spatial resolution of ~20 µm. The comparison of these maps (Fig. 2d) with the regenerated IRPL maps (T_n_; Fig. 2b), suggest a correlation between the potassium-rich (K-rich) areas and the intense IRPL emission areas. Quantitatively, a relationship between IRPL and geochemistry was evaluated by calculating the Pearson’s correlation coefficient (ρ) between signal intensity of any pixel that had detectable IRPL (above background) and the corresponding pixels in the µXRF elemental concentration maps. Table 1 lists the obtained correlation coefficients (all statistically significant with p < 0.01). IRPL intensity positively correlates only with K (ρ = 0.50) and Al (ρ = 0.11). This confirms that the IRPL signal must have a bias towards the K-feldspar end-members, which here contributes to ~60% of the total IRPL from the rock slab. All the remaining elements (including Na and Ca, with ρ = −0.15) had negative correlation with IRPL, suggesting a lack of contribution of IRPL from the Na and Ca feldspar end-members in our sample.

**Table 1:** Pearson’s correlation coefficients between HR-IRPL pixel intensity and corresponding element abundance pixel intensity (determined by µXRF).

Element	Si	Al	K	Na	Ca	Fe	Mn	Mg	Ti	P	C
Pearson’s ρ	−0.05	+**0**.**11**	+**0**.**50**	−0.15	−0.15	−0.25	−0.16	−0.27	−0.13	−0.17	−0.10

All correlations are statistically significant (p-values < 0.01). The only two elements showing a positive correlation with IRPL (Al and K) suggest K-feldspar (KAlSi_3_O_8_) as the primary source mineral for the IRPL emission.

### Luminescence-depth profiles in naturally exposed rock

**Figure 3:**
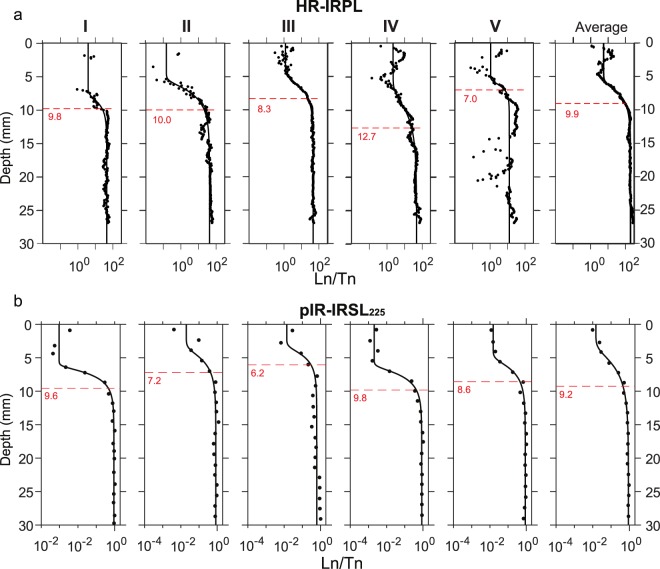
(**a**) HR-IRPL depth profiles derived from the summed pixel rows over the regions I–V as shown in Fig. 2c. (**b**) pIR-IRSL_225_ -depth profiles measured from rock slices from five drilled cores. The bleaching fronts (SD_50%_ value) for the individual profiles in both (**a**,**b**) are plotted as dashed red lines. For both HR-IRPL and pIR-IRSL_225_ the ‘Average’ depth profile (shown on far right) corresponds to an average of all the preceding profiles, with shaded areas showing standard error on L_n_/T_n_. All profiles are fitted with Eq. (1).

**Figure 4:**
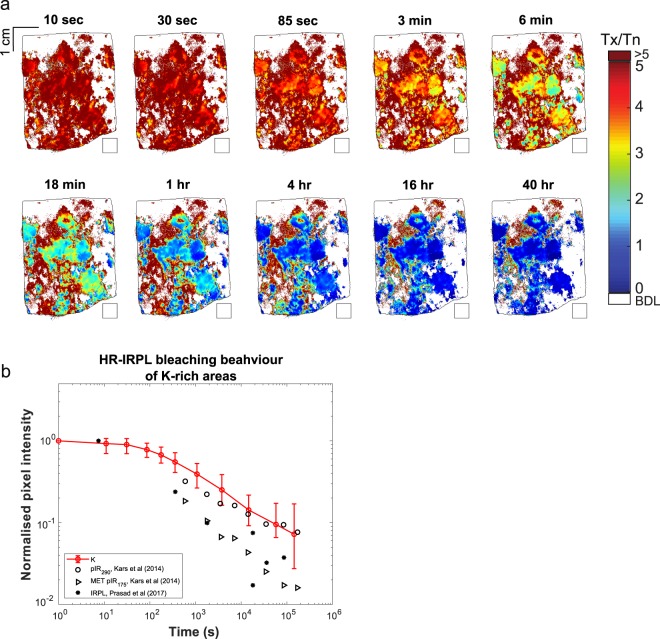
(**a**) False colour maps showing the decrease in T_x_/T_n_ after light exposure of the entire slab in a solar simulator for different durations. The black square below the slab was used for background determination. (**b**) HR-IRPL bleaching curve derived from figure (**a**) after applying a potassium mask and rejecting the values of T_x_/T_n_ ≥ 5. The K- mask used only those pixels which fell within the 95^th^ quantile of K-concentration distribution, in order to preferentially select the K-feldspar end member regions. The median value from the masked T_x_/T_n_ maps is then plotted a function of exposure time. Error bars represent 1σ. K-feldspar bleaching data for IRPL^38^, and pIR-IRSL_290_ and MET pIR_175_ signals^54^ is shown for comparison.

The five IRPL-depth profiles extracted from the L_n_/T_n_ maps (marked as I–V on Fig. 2c,d), and the five pIR-IRSL_225_ depth profiles from the coring-slicing technique are shown in Fig. 3a,b. The dashed lines show the bleaching front, i.e. the 50% saturation depth (SD_50%_)^11^ values, based on fitting with the double exponential equation^10^ (Eq. (1) in section 6). The IRPL-depth profiles (Fig. 3a) show some scatter in the shallowest parts. In profile I and II only a few L_n_/T_n_ ratios are registered in the shallow part after the filtering based on the luminescence intensity threshold; this is due to the absence of IRPL emitting phases near the surface in I and II. In the profiles IV, V and the average profile (ΣL_n_/ΣT_n_) there is a tendency for L_n_/T_n_ to rise from the surface to a depth of ~2 mm, followed by a decrease. The shallow regions with high such ratios correlate with low K-content (Fig. 2d) and seem to emit dim IRPL that is relatively insensitive to the applied dose (Fig. 4, discussed later); this insensitivity to dose results in a similar value for L_n_ and T_n_ and thereby high L_n_/T_n_ ratios near the surface (see Appendix A.2. for the individual L_n_ and T_n_ values). The solid black curves show the model fits (Eq. 1) to the individual IRPL-depth profiles, with µ and $\overline{\sigma {\phi }_{0}}\,t$ treated as independent model parameters (i.e., non-shared across the fits). All the parameter values obtained by fitting are listed in Table 2. Values of the attenuation coefficient (µ) vary between 0.33–1.2 mm^−1^ (average 0.68 ± 0.15), for the five profiles, and the time integrated detrapping constant $(\overline{\sigma {\phi }_{0}}\,t)$ varies by two orders of magnitude from 10^1.7^ to 10^3.5^. These values of the attenuation coefficient are broadly consistent with values reported in the literature^1,10,25,53^. The HR-IRPL SD_50%_ depths from the five profiles fall between 7 mm (profile V) and 12.7 mm (profile IV), with an average of 9.6 ± 0.9 mm; this value is similar to the value of 9.05 mm obtained from fitting (Eq. 1) of the average profile (last column in Fig. 3a).

**Table 2:** Best-fit parameters from Eq. (1) for the HR-IRPL and pIR-IRSL depth profiles (Fig. 3), alongside the corresponding interpolated SD_50%_ depths.

	HR-IRPL profiles						pIR-IRSL_225_ profiles					
	I	II	III	IV	V	AP*	1	2	3	4	5	AP*
*Best-fit parameters (*Eq. 1*):*												
Time-integrated detrapping constant, $\overline{\sigma {\phi }_{0}}\,t$	10^3.23^	10^2.02^	10^1.90^	10^1.67^	10^3.49^	10^2.18^	10^2.81^	10^1.48^	10^1.64^	10^2.56^	10^1.95^	10^1.23^
Luminescence at saturation, L0	40.65	41.81	45.56	45.68	11.56	187.08	0.91	0.83	0.61	0.86	0.88	0.87
Residual luminescence, L_res_	0.09	0.00	0.03	0.05	0.09	0.03	0.00	0.02	0.01	0.00	0.02	0.01
Attenuation coefficient, µ (mm^−1^)	0.80	0.50	0.57	0.33	1.20	0.60	0.72	0.52	0.67	0.64	0.57	0.35
Interpolated parameters:												
Saturation half-depth, SD_50%_ (mm)	9.8	10	8.3	12.7	7.0	9.1	9.6	7.2	6.2	9.8	8.6	9.2

*AP stands for the profile derived from the average of the other profiles (see Fig. 3).

The low resolution pIR-IRSL_225_ depth profiles from the sliced cores (see methods section), measured on a Risø TL/OSL reader (Fig. 3b), show lesser scatter in the L_n_/T_n_ ratios, µ (average 0.62 ± 0.08 mm^−1^; range 0.25–0.72 mm^−1^) and $\overline{\sigma {\phi }_{0}}\,t$ (range 10^1^–10^3^) values compared to the IRPL-depth profiles. Nevertheless, both the average SD_50%_ depth of the five profiles (8.3 ± 1.6) and the SD_50%_ depth of the average profile (9.2 mm) are similar to those from the HR-IRPL (Table 2); this suggests that the HR-IRPL bleaches at a similar rate as the pIR-IRSL_225_ signal in nature.

The model fits suggest that that residual luminescence values at the surface (L_res_) range from 0 to 0.09 (Table 2) with an average of 0.05 ± 0.02. The L_res_ values for pIR-IRSL_225_ profiles range from 0 to 0.02 with an average of 0.01 ± 0.005. Since the surface is known to be light-exposed for ~11 ka^39^, the slightly higher residual values are likely artefacts of the approach adopted for background signal subtraction (see section 6), and the presence of insensitive minerals giving rise to high HR-IRPL L_n_/T_n_ ratios near the surface, as discussed earlier. Nonetheless, to confirm the zeroing of HR-IRPL during light exposure, we designed measured bleaching as detailed below.

### Reduction in IRPL intensity by controlled light exposure

We investigated on the bleaching behaviour of the HR-IRPL by delivering a 2.5 kGy dose to the rock slab (to saturate all the traps) and thereafter exposing it to a solar simulator (artificial light similar to the solar spectrum) for different durations ranging from 10 seconds to 40 hours. The HR-IRPL images were measured immediately following the gamma irradiation (T_n_) and after each subsequent light exposure (T_x_). Figure 4a shows the HR-IRPL images derived from the ratio T_x_/T_n_. The IRPL signals in the K-feldspar regions start to noticeably decrease after a few minutes and reache their observed minimum values by the end of 40 hours of cumulative bleaching (Fig. 4a). Interestingly, the top 5 mm in all the bleaching maps (e.g. see 1 hr duration) show T_x_/T_n_ > 1; this is unexpected since IRPL should reduce due to exposure to the solar simulator^38^ and not increase or remain constant. We suspect that the IRPL intensity of the minerals present in this depth range is simply not responding to dose or bleaching; this may be either be real behavioural aspect of IRPL in some minerals or an artefact of inadequate background subtraction. Nonetheless, this seems to explain the apparent high residual L_n_/T_n_ values in the beginning of the IRPL-depth profiles (Fig. 3).

Given the strongest correlation between the K-rich areas and the IRPL intensity, we further isolated the IRPL emitted from K-feldspars based on the μ-XRF maps and plotted their integrated residual IRPL as a function of bleaching time (Fig. 4b). These data are plotted alongside the IRPL bleaching data from a K-feldspar sediment extract reported by Prasad, *et al*.^38^, and the pIR-IRSL_290_ and MET pIR-IRSL_175_ signals from sand-sized K-rich feldspars reported by Kars *et al*.^54^. The IRPL signal from our rock slice bleaches at a similar rate to the pIR-IRSL_290_ signal, and only 7% of the signal remains after 40 hours of bleaching in the solar simulator. The data from Prasad, *et al*.^38^ seems to follow the pIR-IRSL_175_ data. The difference between our IPPL bleaching curve and that obtained by Prasad *et al*.^38^ may be attributed to: (a) slower bleaching in thick rock slices compared to sediment grains of about 90–180 μm used by Prasad *et al*.^38^ and (b) use of an 830 nm laser in contrast to 885 nm laser used by Prasad *et al*.^38^. We suspect that using a shorter wavelength laser results in a contamination from the second IRPL emission at 880 nm, recently reported by Kumar *et al*.^55^ into the 955 nm IRPL emission targeted in our study. The 880 nm emission is relatively more difficult to bleach than the 955 nm IRPL emission^55^ and this Stokes-shifted emission was likely preferentially suppressed in Prasad *et al*.^38^ as they used a lower energy excitation at 1.40 eV (885 nm) compared to 1.49 eV (830 nm) used in the current study. Nonetheless, these measurements confirm that the IRPL signal from rocks is bleachable by daylight and therefore, the higher L_n_/T_n_ ratios deduced for the surface slices are likely an artefact of the analytical technique.

## Discussion

The coring-slicing method used for the conventional RSD measurements, here using pIR-IRSL_225_, resulted in a loss of up to 11% mass from the cores; this loss fundamentally limits the resolution of the luminescence depth profile. The average random error in slice depths was 0.7 mm which, when applied to a modelled depth profile of a 10 ka exposure using Eq. (1) (see methods), introduces a ~15% uncertainty in the exposure age, regardless of the luminescence data quality. Contrary to this, the sample preparation and high-resolution IRPL imaging of the rock slab was rapid and efficient, with no sample breakage or material loss needing to be accounted for, and much smaller depth uncertainties (detection of the slab edge may be erroneous by one pixel at most, i.e. 140 µm). Sample preparation took only ~15 minutes, and measurement time was reduced to a few minutes for HR-IRPL imaging of one rock slab, compared to the preparation time of ~6 hours, followed by another ~13 hours of reader time for measurement of one core using pIR-IRSL_225_ (with 15 slices).

As expected, because of the high signal sensitivity the IRPL images were sharp with high contrast boundaries between the IRPL emitting and non-emitting areas. In comparison, the SR-IRSL image (measured after a 2.5 kGy dose, Appendix A.1) resulted in relatively dimmer signals and blurrier mineral boundaries. With the integration times used here, there was a twenty-fold increase in intensity in the regenerated HR-IRPL (T_n_) compared to the SR-IRSL image; use of longer integrational times are expected to result in proportionately higher counts and improved signal-to-noise ratio, because of the steady state nature of the IRPL (Fig. 1c). The brightest HR-IRPL and SR-IRSL signals both clearly arise from K-rich feldspar areas (compare Fig. 2 and A.1). This spatial correlation, as well as the similar bleaching rate of K-feldspar HR-IRPL to that of K-feldspar pIR-IRSL_290_^54^ supports the findings of Prasad *et al*.^38^, who saw a tendency for brighter IRPL from K-feldspar. The presence of IRPL in the top 0.5 mm Na rich area (Profile IV in Fig. 2b,d), support the findings of Prasad *et al*.^38^, that Na-rich feldspars also emit IRPL. However, our data shows negative correlation coefficient (Table 2) between Na content with IRPL, likely suggesting that K is replacing sodium in some phases (e.g. in alkali feldspar). However, we cannot rule out the possibility that the IRPL may actually arise from K-rich feldspar below the surface. Further studies of the penetration depth of the IR laser and transmission of the IRPL signal through the rock will be needed to draw firm conclusions. We see no correlation between quartz and IRPL; this is not surprising since IR stimulatable signal from quartz has only been observed at elevated temperatures^56,57^ in the UV emission region^58,59^. The Ca-rich areas generally correspond to mica in our rock slab; these show negative correlation with IRPL. Although micas have been reported to emit luminescence during IR stimulation, their relative contribution compared to the feldspar IRSL has not been fully addressed^60,61^.

A recent study by Meyer *et al*.^25^ investigated the effects of mineral heterogeneity on light penetration into various rock types (including a granitic gneiss with mineral size of ~10 mm). A correlation was found between rock slice opacity and local L_n_/T_n_ maxima/minima due to ‘shadowing’ effects from opaque minerals. Considering the large (≤10 mm) crystal size in our rocks sample it is conceivable that spatial mineral heterogeneities may also result in scatter in our data and an inter-profile variance in µ and SD_50%_. The pIR-IRPL_225_ profiles show smaller variance in µ and $\overline{\sigma {\phi }_{0}}\,t$ across the five profiles compared to the HR-IRPL; this may suggest that either all our 10 mm diameter cores had comparable spatial distributions of minerals, or the effect of mineralogical heterogeneity was averaged out at the cost of lower profile resolution.

A major accomplishment of HR-IRPL is a ten-fold increase in the profile resolution compared to the conventional coring-slicing method. Nonetheless, the HR-IRPL profiles I, II and V still have few L_n_/T_n_ points in the shallower part of the profile (i.e. <5 mm) since not all the crystals emit IRPL in our rocks. The scatter in the profile may result from optical heterogeneities, slight misalignment of the L_n_ and T_n_ pixels, or possible differences in IRPL behaviour across different regions; as discussed earlier, IRPL in some regions of the rock do not seem to respond to gamma irradiation or solar bleaching, thereby potentially contributing to the scatter (high L_n_/T_n_ ratio) in the shallow regions of the profiles. Since these are exposure (and not burial) profiles for a duration of ~11 ka, we expect bleaching rather than trapping^62,63^ to be the dominant factor governing luminescence intensities; thus, it is unlikely that scatter can be attributed to possible dose rate variation in the shallow regions of the profiles. It remains to be seen in future studies if extending the measurement duration results in more complete profiles because of the better counting statistics from the less sensitive regions. The HR-IRPL RSD may further benefit from improved methods for background subtraction, e.g. by determining spatially resolved background values on the slab after complete bleaching.

The overlap of the average SD_50%_ depths between the HR-IRPL and pIR-IRSL_225_ profiles, suggest similar bleachability of the two signals. However, in contrast to pIR-IRSL, IRPL saves sample preparation and measurement time, avoids issues with thermal lag, sensitivity change and loss of precision due to slicing, and finally presents an opportunity to correlate luminescence and µXRF maps. Furthermore, it may be possible to derive HR-IRSL profiles by calculating a ‘difference’ image between IRPL before and after an extended IR illumination. This raises the possibility of simultaneous, multiple-signal (IRSL and IRPL), high-resolution imaging in RSD; this may be useful for dating young or rapidly-eroding surfaces^1^ since IRSL is more readily bleachable than IRPL and post IR-IRSL signals. The main challenges in the application of HR-IRPL as presented here are the need for an ioinsing radiation source (e.g., gamma emitter) providing uniform irradiation of the whole rock slab, accurate background subtraction, and accurate pixel alignment between the L_n_ and T_n_ images. The issue with ionising radiation source is the most critical; future work could explore normalisation using different signals (e.g. IRSL, 880 nm IRPL, or non-bleachable background) to compensate for the varying spatial sensitivities, rather than using the response to a test dose from an ionisation radiation source.

## Conclusions and Outlook

High-resolution spatially resolved infrared photoluminescence (HR-IRPL) has been developed for direct mapping of trapped electron populations in large rock slabs. We demonstrate its potential application for rock surface dating by mapping the luminescence-depth profile in a slab of exposed granite. The new HR-IRPL technique is benchmarked against the conventional pIR-IRSL_225_ technique; both methods resulted in similar optical bleaching fronts, but larger scatter was observed in HR-IRPL data. Further work is necessary to understand the sources of scatter in HR-IPRL depth profiles, in particular the effect of dim minerals and the background on the precision and accuracy of the sensitivity corrected images. In contrast to the conventional RSD, the new method involved minimal sample preparation and measurement time, higher profile resolution, and negligible depth uncertainty. Furthermore, IRPL images can be correlated with high resolution geochemical maps; here the correlation with µXRF data suggests that K-feldspar is the primary emission phase for IRPL.

Since the HR-IRPL method is based on direct measurement of trapped electron concentrations in the rock samples, it has the potential to (a) further test and refine our mathematical models of rock surface dating and calibration procedure, and (b) significantly enhance our understanding of light propagation in natural minerals and rocks at sub-micrometer resolution. Given the simplicity of instrumentation, our study opens possibilities for future development of a portable IRPL imaging instrument. With appropriate sensitivity normalisation, a non-destructive IRPL measurement in the field may be used for guiding sample selection for both exposure and burial RSD. The ease and efficiency of HR-IRPL data collection will support the development of novel applications in quantitative geomorphology and archaeology.

This study marks the new era of high precision, spatially resolved luminescence dating of natural minerals with the potential to give a significant thrust to Quaternary geosciences on recent and prehistoric timescales.

## Materials and Methods

### Sample selection and preparation

**Figure 5:**
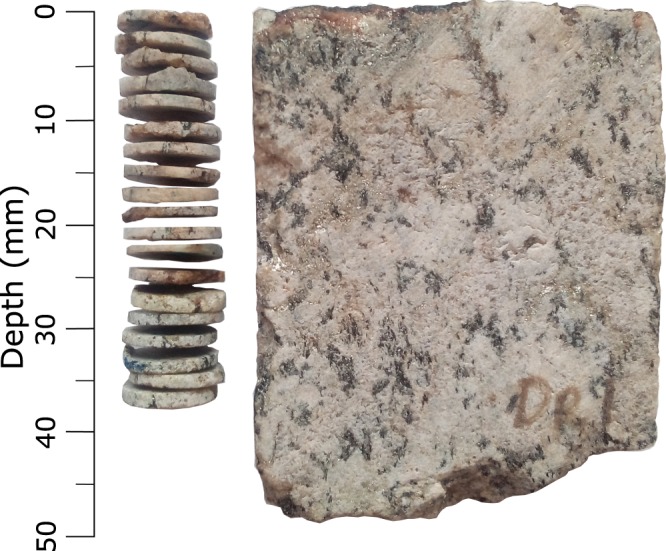
Photograph of a sliced rock core used for conventional RSD measurements (left). Photograph of the rock slab used for the HR-IRPL measurements (right).

For our target material, we resampled a previously studied and known-age location^39^ (got-11), consisting of a well-preserved glacially polished surface from the Gotthard Pass, Switzerland. The granitic bedrock at the site is ~300 Ma old^64^, with abundant large (≤10 mm) white feldspar and quartz crystals. The current rock surface was sub-aerially exposed ~11 ka ago during the post-Late Glacial Maximum (LGM) retreat^39^. Many rock surfaces at the pass, including ours, still retain mirror-like glacial-polish^65^, indicating negligible surface modification (through erosion or weathering) since the LGM deglaciation. Blocks of ~10 cm^3^ were collected from the granite using a petrol-powered saw and stored in lightproof conditions. One block was further cut into ~3 × 5 × 1 cm slabs (Fig. 5), perpendicular to its original surface (by aligning the flat glacially polished exposed surface at 90° with the cutting blade), using a large water-cooled diamond saw under subdued orange light^66^. One of these slabs was used directly (without polishing) for the HR-IRPL measurements reported here. Five cores (ø ≅ 1 cm) from two other blocks were also drilled perpendicular to the blocks’ surfaces, and further sliced into ~1.3 mm thick slices using a water-cooled precision saw (Fig. 5). The desired slice thickness was mechanically regulated using an analogue micrometre, by advancing the core holder exactly 1.5 mm towards the diamond blade. After slicing, triplicate thickness measurements were made of each individual slice, and of the length of the remaining core using a digital calliper. These three measurements (micrometre, slice and core thickness) were then averaged to calculate the depth of each slice from the surface of the rock.

### Instrumentation

For pIR-IRSL measurements, the rock slices were placed directly onto the sample carousel and measured on a standard Risø OSL/TL TL-DA-20 reader, with a ^90^Sr/^90^Y beta source, IR light emitting diodes (~ 870 nm, 1.43 eV) for stimulation, and a blue filter pack comprising a combination of Schott BG-39 and Corning 7–59 (320–480 nm) filters for luminescence detection^67^. Each slice was preheated to 250 °C for 100 s, and then optically stimulated using IR LEDs for 200 s, first at 50 °C (IRSL_50_) and subsequently at 225 °C (pIR-IRSL_225_), to record the natural L_n_ signals (Fig. 1b). The regenerated luminescence following a 20 Gy test dose (T_n_) was measured in a similar manner. Only the pIR-IRSL_225_ emission is discussed in this study, as it’s bleaching behaviour in the natural sample was observed to be similar to the IRPL signal^38^.

For the HR-IRPL imaging, the surface-perpendicular granite slab was placed on a small sample stage directly under an Evolve 512 OEM EM-CCD camera. The camera was fitted with 2 × 925 nm long-pass interference filters and a 950 Δ50 nm bandpass filter (optical density, OD:4). The sample stage was illuminated with a 1020 nm IR LED array to check sample’s position between HR-IRPL imaging. The sample was excited with 830 nm (1.49 eV) laser cleaned with an 850 nm short-pass filter (optical density, OD:4) to measure the HR-IRPL emission (Fig. 1d). The laser was defocussed to cover the entire sample with a uniform power density of 0.2–0.6 mW.cm^−2^. Laser stimulation is necessary to avoid excitation light breakthrough into the detector, due to the proximity of the stimulation and emission wavelengths (see Fig. 1b,c); for this reason IR LEDs cannot be used for stimulation for measurement of IRPL. Note that our excitation wavelength differs from Prasad *et al*.^38^ who used an 885 nm (1.40 eV) laser. We chose a shorter wavelength, although still consistent with the excitation spectrum of IRPL^38^, as it was necessary to reduce the breakthrough, and thereby achieve a high signal-to-noise ratio necessary for high resolution IRPL mapping. The granite slab was pre-heated on a hot plate for 5 minutes at ~230–250 °C before the acquisition of both the natural (L_n_), and the 2.5 kGy saturating test dose (T_n_) HR-IRPL images. The test dose was delivered in a Cobalt-60 gamma cell facility at DTU Nutech High Dose Reference Laboratory (dose rate of ~3.98 Gy/min), and was aimed at bringing all dosimetric traps to saturation. The HR-IRPL images were acquired at room temperature with a 1 second integration period, the pixel intensity being recorded as a 2-byte greyscale image. Although longer integration times could be used, we found that reasonable counting statistics were achieved in this 1 second integration window. To determine the bleachability of the HR-IRPL signal under controlled laboratory conditions, the granite slab was irradiated with another 2.5 kGy gamma dose after the IRPL T_n_) measurement, and then subjected to incremental bleaching durations ranging between 10 seconds and 48 hours in a Hönle SOL 2 solar simulator. The surface used for HR-IRPL imaging was exposed to the light to ensure uniform bleaching of the entire slab, and the HR-IRPL emission (T_x_) was recorded after each succeeding bleaching. No pre-heat was used before measurement in order to more closely simulate the natural bleaching processes in nature. After completing the bleaching experiment, the granite slab was gamma-irradiated again with 2.5 kGy, and the spatially-resolved IRSL (SR-IRSL) was recorded over a 100-second period, using the same stimulation source as for HR-IRPL, but detected through a BG-39 and BG-3 filter pack fitted onto the EM-CCD camera. This image was collected over a longer time period due to the IRSL signal being much dimmer than the IRPL. The image was used for comparison with the HR-IRPL. Finally, quantitative maps of the major oxides found in granites (Si, Al, Ca, Na, K, P, C, Mg, Mn, Fe, and Ti) were obtained using an M4 Tornado 2D µXRF scanner, at DTU Nutech, at a 0.1 mm/pixel resolution. As the main mineralogical constituents of the Gotthard granite are quartz (Si-rich) and feldspar (with potentially K, Na and Ca end-members), the presence of Si, Al, K, Na and Ca was considered directly relatable to the presence of quartz and feldspar end-members^68,69^.

### Statistical methods

Due to slight variation in the placement of the granite slab on the sample stage under the EM-CCD camera during each imaging, the natural (L_n_) and test dose (T_n_) HR-IRPL images required spatial alignment. We used an affine image registration (imregister) in MATLAB’s Image Processing Toolbox^70^ to achieve the alignment. The array of HR-IRPL bleaching images (T_x_) was aligned onto the master T_n_ HR-IRPL image in a similar fashion. On each HR-IRPL image, a fixed region corresponding to a 40 × 40 pixel area outside of the rock slab, was selected as representative of the background emission; the value of the highest-intensity pixel in this area was then subtracted from all image pixels, with values < 0 tagged as “below detection limit” (BDL) and removed. The natural HR-IRPL intensities were divided by the test dose intensities (L_n_/T_n_). Five equal sub-sections were defined across the width of the slab, and due to the skewed distribution of intensity values per row, the summed intensity values from each pixel row were used to construct five adjacent depth profiles. An average HR-IRPL L_n_/T_n_ profile was calculated by summing all pixel values across each row along the whole width of the rock slab from the individual L_n_ and T_n_ images. An averaged pIR-IRSL225 profile (averaging all L_n_/T_n_ values from the five profiles) was also made. All depth profiles were fitted with the double exponential model, Eq. (1) ^10^:

1 $$L(x)={L}_{0}{e}^{-\overline{\sigma {\phi }_{0}}t{e}^{-\mu x}}+{L}_{res}$$

The model is suitable for relatively short exposure durations and discounts the effects of dose rate. *L(x)* is the luminescence at depth *x* (mm), *L*_0_ is the maximum luminescence at signal saturation, and *L*_*res*_ the residual and/or unbleachable luminescence component. The two kinetic parameters in Eq. (1), governing the shape and depth of a luminescence profile, are the light attenuation coefficient *µ* (mm^−1^), and the composite parameter $\overline{\sigma {\phi }_{0}}\,t$ (unit-less), representing the detrapping constant at the rock’s surface $\overline{\sigma {\phi }_{0}}$ (ka^−1^) averaged over its cumulative exposure time *t* (ka)^9^. After fitting each individual profile, the 50% saturation depths (SD_50%_, corresponding to the depth *x*, at which *L(x)* = 0.5*L*_0_)^11^ were calculated, and used as a baseline metric for comparing the luminescence bleaching front across all profiles.

Because of the different spatial configurations of the acquired images from the flatbed-scanning µXRF and the EM-CCD camera set-up, the quantitative µXRF elemental maps required manual image alignment and registration onto the background subtracted T_n_ image, using the BigWarp tool from ImageJ^71^. Thereafter, the two aligned images were used calculate the Pearson correlation coefficients between the IRPL intensity and the elemental concentration pixels.

To evaluate the bleaching behaviour of the IRPL emission in K-rich feldspar, the pixels with highest K intensities (95^th^ quantile) were identified from the K-map; the corresponding pixels were then identified in HR-IRPL bleaching images (T_x_). The bleaching curve of the IRPL signal was obtained by taking the median HR-IRPL intensity from relevant pixels in each T_x_ image and plotting it as function of exposure time.

## Supplementary information

Appendix

## Data Availability

The datasets generated during and/or analysed during the current study are available from the corresponding author on reasonable request.
