# Supplementary material for: Optical bleaching front in bedrock revealed by spatially-resolved infrared photoluminescence
Source: Sci Rep. 2019 Feb 22;9:2611. doi: 10.1038/s41598-019-38815-0 (PMC6385230; doi:10.1038/s41598-019-38815-0)
Supplement: Supplementary file 1 — Appendix [file 41598_2019_38815_MOESM1_ESM.pdf]

Optical bleaching front in bedrock revealed by spatially-resolved infrared photoluminescence

Sellwood, E.L., Guralnik, B., Kook., M., Prasad, A.K., Sohbati, R., Hippe, K., Wallinga, J., Jain, M.

Appendix

A.1

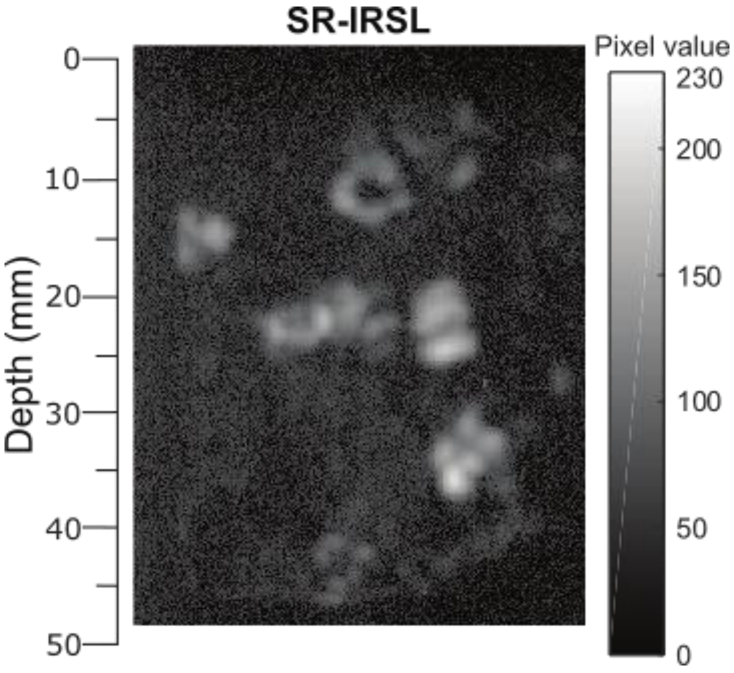

Appendix A.1. The SR-IRSL signal after 2.5 kGy dose. It shows that the maximum pixel intensity is significantly lower than that of the HR-IRPL (Fig.3b).

A.2

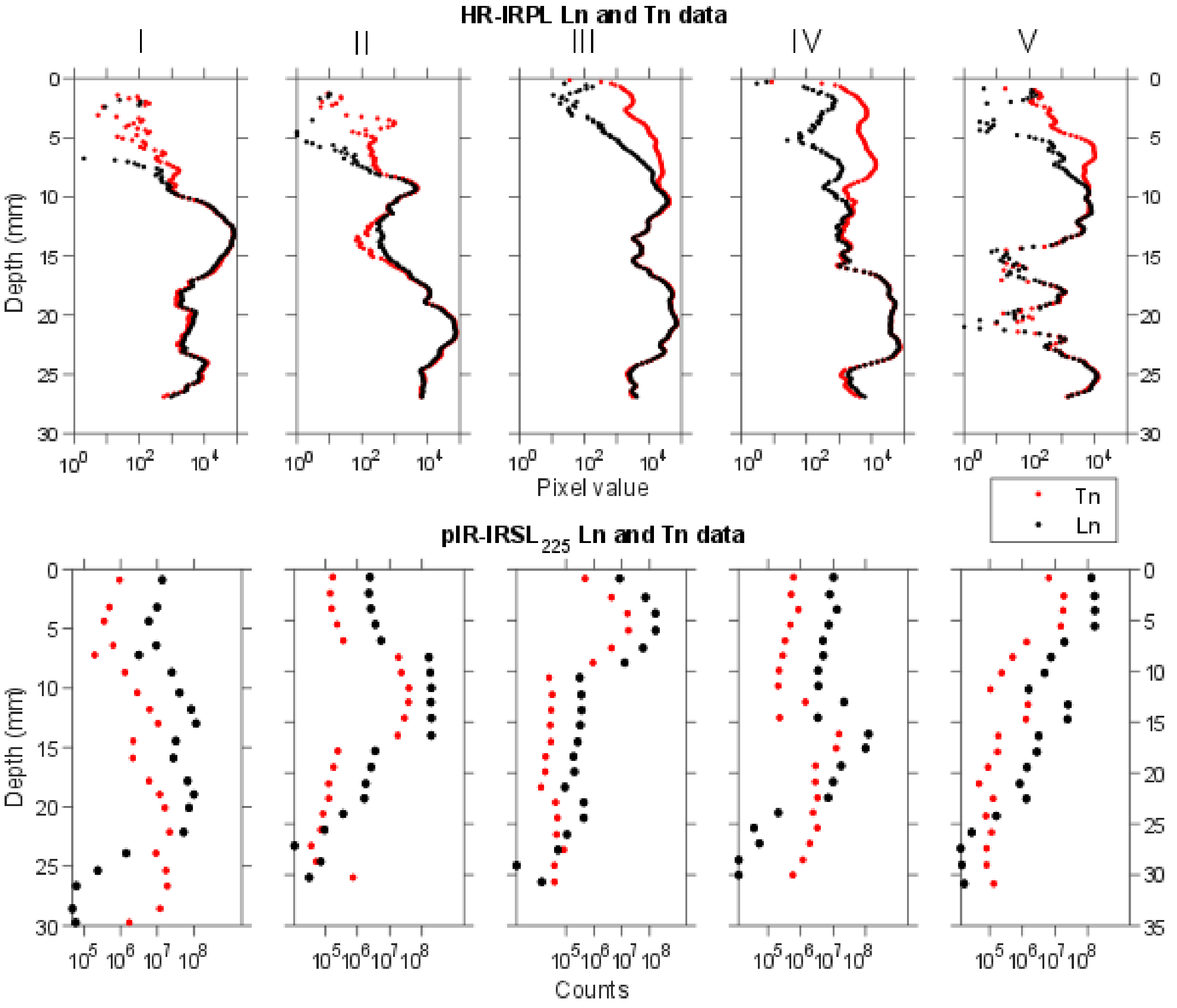

A.2 Appendix A.2. The natural (L_n, black) and regenerated (T_n, red) data for the HR-IRPL and pIR-IRSL$_{225}$ depth profiles, presented on log scale.

A.3

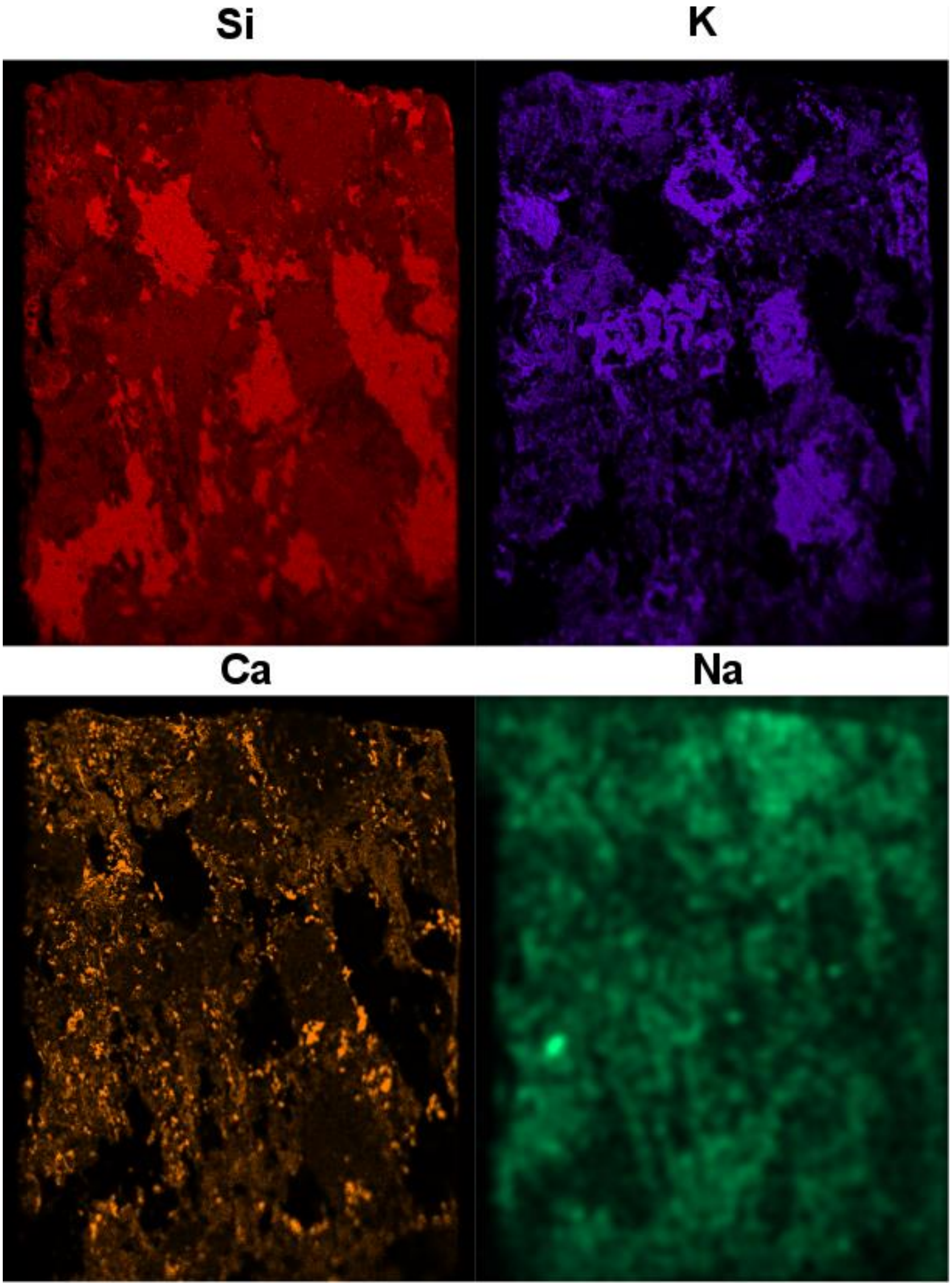

Appendix A.3. Individual elemental maps for Si, K, Ca and Na. Note the colours for the elements are different to those in the composite elemental map shown in figure.2.
